# Involvement of HDAC6 in ischaemia and reperfusion-induced rat retinal injury

**DOI:** 10.1186/s12886-018-0951-7

**Published:** 2018-11-20

**Authors:** Haihong Yuan, Hui Li, Ping Yu, Qichen Fan, Xuan Zhang, Wei Huang, Junyi Shen, Yongyao Cui, Wei Zhou

**Affiliations:** 1 Department of Pharmacy, Shanghai University of Medicine & Health Science, Shanghai, China; 2 Department of Pharmacology, Shanghai Jiao Tong University School of Medicine, 280 South Chongqing Road, Shanghai, 200025 China; 3 Department of Pharmacy, Qingpu Branch of Zhongshan Hospital, Fudan University School of Medicine, Shanghai, China; 4 Laboratory of Oral Microbiota and Systemic Diseases, Shanghai Research Institute of Stomatology, Ninth People’s Hospital, Shanghai Jiao Tong University School of Medicine, 115 Jin Zun Road, Shanghai, 200125 China; 5 Shanghai Key Laboratory of Stomatology & Shanghai Research Institute of Stomatology, National Clinical Research Center of Stomatology, Shanghai, China

**Keywords:** Glaucoma, Ischaemia and reperfusion, Neuroprotection, HDAC6, Tubacin

## Abstract

**Background:**

The role of histone deacetylases 6 (HDAC6) has been elucidated in various neurodegenerative diseases. However, the effect of HDAC6 on retinal degenerative processes remains unknown. The aim of this study was to elucidate the potential role of HDAC6 in the retinal ischaemia and reperfusion (I/R) injury model.

**Methods:**

The retinal pathological lesion was evaluated by haematoxylin and eosin (H&E) staining. HDAC expression or activity was detected by immunohistochemistry, Western blotting assays or colorimetric assays. The expression of apoptotic- and autophagic- related proteins were quantified by Western blotting and RT-PCR. The expression of peroxiredoxin 2 (Prx2) was determined by RT-PCR and ELISA. The levels of acetylated α-tubulin and acetylated histone 3 in the retina were assayed by Western blotting.

**Results:**

We found that I/R-induced reduction of the retinal thickness was ameliorated, and the survival of RGCs was increased by the histone deacetylase (HDAC) inhibitor Trichostatin A (TSA) as well as by tubacin (an HDAC6 selective inhibitor). The decreased expression of THY (thymus cell antigen) in the I/R-induced retinas was also reversed by TSA and tubacin. Elevated HDAC6 expression and activity in the retina from I/R injury were significantly inhibited by tubacin, which also attenuated I/R-mediated apoptosis by decreasing TUNEL-positive RGCs and Bax expression and increasing Bcl-2 expression. Additionally, tubacin increased the expression of autophagy-related gene Beclin 1 and microtubule-associated protein 1 light chain 3B (LC3B) and the levels of Prx2. Furthermore, the protective effect of tubacin was associated with acetylated α-tubulin and was independent of acetylated histone 3.

**Conclusions:**

Our findings suggest that tubacin exhibits neuroprotective effects after I/R retinal injury, and HDAC6 may be a potential therapeutic target for the retinal neurodegenerative disease of glaucoma.

## Background

Glaucoma is recognized as a collection of neurodegenerative diseases that result in retinal ganglion cell (RGC) degeneration and death [1]. Intraocular pressure (IOP) is regarded as the major risk factor. This is a multifactorial disease, and although intraocular hypertension remains an important risk factor, glaucomatous damage results from a combination of intraocular hypertension and IOP-independent risk factors [2]. Despite efforts to understand the pathological processes in the retina, few prophylaxis treatments are available to prevent glaucoma.

Retinal ischaemia/reperfusion (I/R) injury is a common clinical condition that represents the main cause of visual impairment and blindness [3]. In addition to glaucoma, retinal ischaemia likely contributes to the aetiology of many retinal diseases, such as retinal artery occlusion, optic neuropathy and diabetic retinopathy [4]. In these situations, retinal I/R results in the dysfunction or death of retina cells and retinal degeneration [5, 6]. An animal model of retinal I/R injury, which mimics acute glaucoma, is continually used to study RGC dysfunction or loss following ischaemic injury [7].

Much evidence has indicated that I/R injury is improved via the modulation of anti-inflammatory factors and pro- and anti-apoptotic and upregulation of heat-shock proteins [8, 9]. Recent researches on retinopathy-induced epigenetic changes have advised new therapeutic methods [6, 10–12]. Epigenetic modifications have suggested a promising new approach to modulate cell function as observed in neurodegenerative diseases [13]. Histone deacetylases (HDACs) have been regarded as a therapeutic target in different diseases from neurodegeneration to cancer [14]. HDACs are enzymes that deacetylate lysine residues on histones as well as on several other cytoplasmic mitochondrial and nuclear non-histone proteins [15]. HDACs assure the reversible acetylation of histones and play an essential role in histone metabolism and transcriptional regulation. Considering the importance of HDACs to biological and pathophysiological processes in neurodegeneration and cancer, various medicinal chemistry studies have developed HDAC inhibitors to treat cancer and other malignant tumours [16, 17].

Recently, scientists studying neurodegeneration have devoted attention to HDACs, especially to HDAC6, because it could be a crucial player in various neurodegenerative diseases. HDAC6, a unique class IIb HDAC, plays an essential role in protein quality control because of its unique characteristics [16]. First, HDAC6 involves two functional N-terminal catalytic sites that are combined with a ubiquitin-binding domain at the C-terminus. Second, HDAC6 is located in the cytoplasm, indicating that its activity neither depends on histone nor influences transcriptional processes [16]. After injury to neurons, the expression of HDAC6 is elevated. Genetic and pharmacological methods have demonstrated that HDAC6 inhibition can promote the survival and regeneration of injured neurons, suggesting that HDAC6 acts as a target for the protection and regeneration in neurodegenerative disorders [18]. However, the effect of HDAC6 on the retina remains largely unknown.

Few reports exist on the effect of HDAC inhibition on RGCs. Crosson et al. [19] and Fan et al. [20] demonstrated that the inhibition of HDAC protected the retina from ischaemic injury. HDAC is involved in the neuroprotection of valproic acid (VPA) on RGCs [6, 21–23]. However, they could not verify changes in the histone acetylation levels. The interpretation of this discrepancy is that VPA-induced neuroprotection may be independent of histone acetylation [24]. Considering a histone acetylation-independent mechanism, HDAC6 could play a crucial role in the neuroprotection provided by HDAC inhibitors in the retina. Therefore, it greatly interested us whether the HDAC6 inhibitor has neuroprotective properties and its mechanism of action in a glaucoma model.

In the present study we used an in vivo retinal I/R injury model to explore the potential role of HDAC6 in glaucoma. Our results showed that HDAC activity and HDAC6 expression were significantly elevated over baseline in the retina from I/R injury in association with pathological retinal events. The HDAC6 inhibitor tubacin exerted retinal neuroprotective effects that were associated with enhanced autophagy, the inhibition of apoptosis, anti-oxidative stress. The protective effects of tubacin may be independent of acetylated histone 3 and rely on acetylated tubulin. Tubacin is a selective HDAC6 inhibitor, and Trichostatin A (TSA) is a pan-HDAC inhibitor.

## Materials and methods

### Animals

Adult male SD rats, whose weight ranged from 200 to 300 g in the study, were provided by the Department of Laboratory Animal Science of Shanghai Jiao Tong University School of Medicine. The rats were kept under ambient temperature (24 ± 2 °C) with a day-night rhythm of twelve hours and were given free water and food intake. All the experimental protocols were approved by the ethical committee of the Animal Care and Experimental Committee of Shanghai Jiao Tong University School of Medicine. All protocols of the animals were in accordance with the ARVO Statement for the Use of Animals in Ophthalmic and Vision Research. For neuroprotection studies, tubacin (1.33 mg/kg), vehicle or TSA (2.5 mg/kg) was administered to rats by intraperitoneal injection once per day for 6 days (3 days before and after ischaemia) [19, 20].

### Animal model of retinal ischaemia and reperfusion

The animal model of retinal ischaemia/reperfusion was established based on a previously described protocol [5]. Briefly, 3% sodium pentobarbital was intraperitoneally injected into rats for anaesthesia, while 0.5% phenylephrine hydrochloride and 0.5% tropicamide were used for full dilation of pupils. A 30-gauge needle in connection with a vessel filled with saline was applied to the cannulating anterior chamber. The vessel was raised to form a hydrostatic pressure ranged from 110 to 120 mmHg for fifty minutes to increase IOP and induce retinal ischaemia. Each pressure was then returned to normal, and the eye was examined to ensure that retinal blood flow was re-established. The experimental rats were divided into four groups—sham group, I/R plus vehicle group, I/R plus tubacin group, and I/R plus TSA group—with five to eight rats in each group.

### Histopathologic study

The rats were anaesthetized with chloral hydrate and were perfused with physiological saline before sacrifice. The experimental globes were enucleated three days after the ischaemia-reperfusion operation. The eyes were fixed with PBS containing 4% paraformaldehyde and were subsequently washed with PBS. After dissecting foreparts and lens, the eye cups were dewatered and embedded using paraffin and were cut into slices horizontally through optic discs at 3-μm thickness. Haematoxylin and eosin (H&E) were used to stain the slices. In each slice, the layers of the retina apart from the centre of the optic nerve head (ONH) for approximately 1.5 mm were collected as picture data and were written into a disk using an optical microscope in connection with a digital camera. The retinal injuries were evaluated by measuring the quantity of cells in the ganglion cell layer (GCL) and thickness of each layer. The nuclear cells in GCL were quantified every 200 μm, and the average value was applied to determine a typical cell quantity in GCL.

### TUNEL staining

Four-percent paraformaldehyde was utilized to fix freshly isolated retinas, and paraffin was used for embedding. The retina sections were mounted on glass slides and then were de-paraffinized/hydrated for TUNEL staining. The TUNEL assay was performed using the In Situ Cell Death Detection Kit (Roche, Penzberg, Germany) according to the manufacturer’s instructions. The retina slides were permeabilized with 0.1% Triton X-100 and blocked with 3% H_2_O_2_. After incubation with the TUNEL reaction mixture for 60 min at 37 °C in a humid chamber, the slides were incubated in Converter-peroxidase (POD) for 30 min at 37 °C and were stained with diaminobenzidine (DAB) POD substrate. Images were acquired by microscopy.

### Measurement of HDAC6 expression and retinal HDAC activity in the retina

The avidin-biotin-peroxidase complex (ABC) technique and HDAC6 antibody (1:50; Cell Signalling Technology, Beverly, MA, USA) were used to process frozen sections of retinas in the I/R + Vehicle, I/R + tubacin, I/R + TSA and sham groups to conduct immunohistochemical analyses at 4 °C overnight. Subsequently, the slices were incubated with ABC complex and biotinylated rabbit anti-goat IgG purified by affinity at ambient temperature for 1 h. In the experiment, 0.1 M PBS containing 0.5% Triton X-100 was used to dilute all antisera. After DAB staining, images were acquired by microscopy.

To assess the fluctuations in HDAC activity affected by tubacin, the retinae dissected free after euthanization was stored at − 80 °C for subsequent experiments. The retinal lysates were sonicated and centrifuged. The supernatant was used to determine the HDAC activity using a HDAC Activity Colorimetric Assay Kit (BioVision, Mountain View, CA, USA) according to the manufacturer’s instruction. The HDAC activity in the samples was calculated based on a standard curve and was normalized to total proteins in the retinae measured using the BCA protein assay kit (Pierce, Rockford, IL, USA).

### Total RNA extraction and quantitative real-time PCR (RT-PCR)

Total RNA was extracted from the retinae using the DNA/RNA/Protein Isolation kit (Omega Bio-Tek, Norcross, USA), and RT-PCR was conducted using M-MLV Reverse Transcriptase (Invitrogen, Carlsbad, USA). The cDNA sequences obtained from the reverse transcription of three parallel samples were utilized to determine the level of target mRNA quantitatively through qRT-PCR with SYBR® Premix Ex TaqTM using a LightCycler® 480IIsequence detector (Roche, Forrenstrasse CH-6343, Rotkreuz, Switzerland). The RT-PCR primer sequences are listed in Table 1. β-Actin was used as a control. The 20-μl sample contained 10 μl of PCR using the SYBR® Premix Ex TaqTM (TaKaRa Biotechnology, Dalian, China), 8 *p*mol of primer and 1.2 μl of RT reaction. The experiments were triplicated in the LightCycler® 480 Multiwell Plate 96 (Roche, Mannheim, Germany). The cycling parameters consisted of 95 °C for 30 s, followed by 40 cycles of 95 °C for 10 s, 60 °C for 20 s, and 72 °C for 15 s. The relative levels of target mRNA expression were calculated using the 2^−ΔΔCt^ method.

**Table 1:** The RT-PCR primer sequences are listed

Gene name	Primer sequence	
	Forward	Reverse
actin	5’-AATCCTGTGGCATCCATGAA-3’	5’-GGACAGTGAGGCCAGGATAGA-3’
beclin-1	5’-AGGAGTTGGCCTTGGAGGA-3’	5’-CCGCTGTGCCAGATATGGA-3’
lc3	5’-CGTCCTGGACAAGACCAAGTT-3’	5’-GGTGCCTACGTTCTGATCTGTG-3’
bax	5’-GAGCGGCTGCTTGTCTGGAT-3’	5’-CAAGGCAGCAGGAAGCCTCA-3’
bcl-2	5′- GCAGATGCCGGTTCAGGTA-3’	5’-ACGGTGGTGGAGGAACTCTT-3’
Prx2	5’-AGGACTTCCGAAAGCTAGGC-3’	5′- TTGACTGTGATCTGGCGAAG-3’
HDAC6	5’-GCACGCTGTCTCATCCTACCT-3’	5’-CCCGAGTTTTCATCTTTTCTGTG-3’

### Western blotting analysis

Retinae were washed twice in PBS and were lysed in ice-cold RIPA buffer (Beyotime Institute of Biotechnology, Shanghai, China, followed by dispersion ultrasonically. The BCA assay kit (Pierce, Rockford, USA) was utilized to determine the concentration of proteins. The samples were mixed with 0.0125% bromophenol blue and 2.5% β-mercaptoethanol and were boiled for five minutes before being subjected to SDS-PAGE. After transferring the protein blots onto PVDF membranes (Merck Millipore, Billerica, USA), the membranes were incubated at 4 °C overnight with the following primary antibodies: HDAC6 (1:500; SANTA CRUZ Biotechnology, Dallas, USA), Beclin1, LC3, Bax, Bcl2 (1:1000; Cell Signal Technology, Danvers, USA), peroxiredoxin 2 (Prx2, 1:500; Sigma, St. Louis, USA), Acetylated-α-tubulin, α-tubulin (1:500; Beyotime Institute of Biotechnology, Shanghai, China), THY (1:500; Merck Millipore, Billerica, USA) and β-actin (1:1000; Zhongshan Biotechnology, Beijing, China). After incubating the membranes with the appropriate secondary antibody conjugated to horseradish peroxidase (1:8000, Zhongshan Biotechnology, Beijing, China), an enhanced chemiluminescence detection kit (Pierce Chemical, Rockford, IL, USA) was utilized to visualize the protein blots.

### Enzyme-linked immunosorbent assay (ELISA) of Prx2

Rats injected with vehicle, TSA or tubacin 1 h before ischaemia of a single retina, aiming to determine whether Prx2 activity was affected by HDAC inhibition. After I/R, the retina was cut open and immersed into lysing buffer containing proteinase inhibitors and then were stored at − 80 °C. Subsequently, ELISA (Xi’tang Biotechnology, Shanghai, China) was conducted to analyse Prx2 activity using the supernatant from the centrifuged extract of retinae. The total protein of retinae was measured using the BCA protein assay (Pierce Chemical, Rockford, IL, USA) and was applied to normalizing the concentration of prx2 in samples which was calculated based on the standard curve. Prx2 activity can be expressed as the concentration per mg protein sample.

### Statistically analysis of the data

All data were represented as mean ± SEM. One-way analysis of variance (ANOVA) and Dunnett’s post hoc tests were successively applied to result analyses. *P* value less than 0.05 was accepted as the threshold of statistical significance. An image-analysis programme (IPP, Olympus, Japan) was used to perform all measurements.

## Results

### Effect of tubacin on retinal morphological injury after I/R injury

**Fig. 1:**
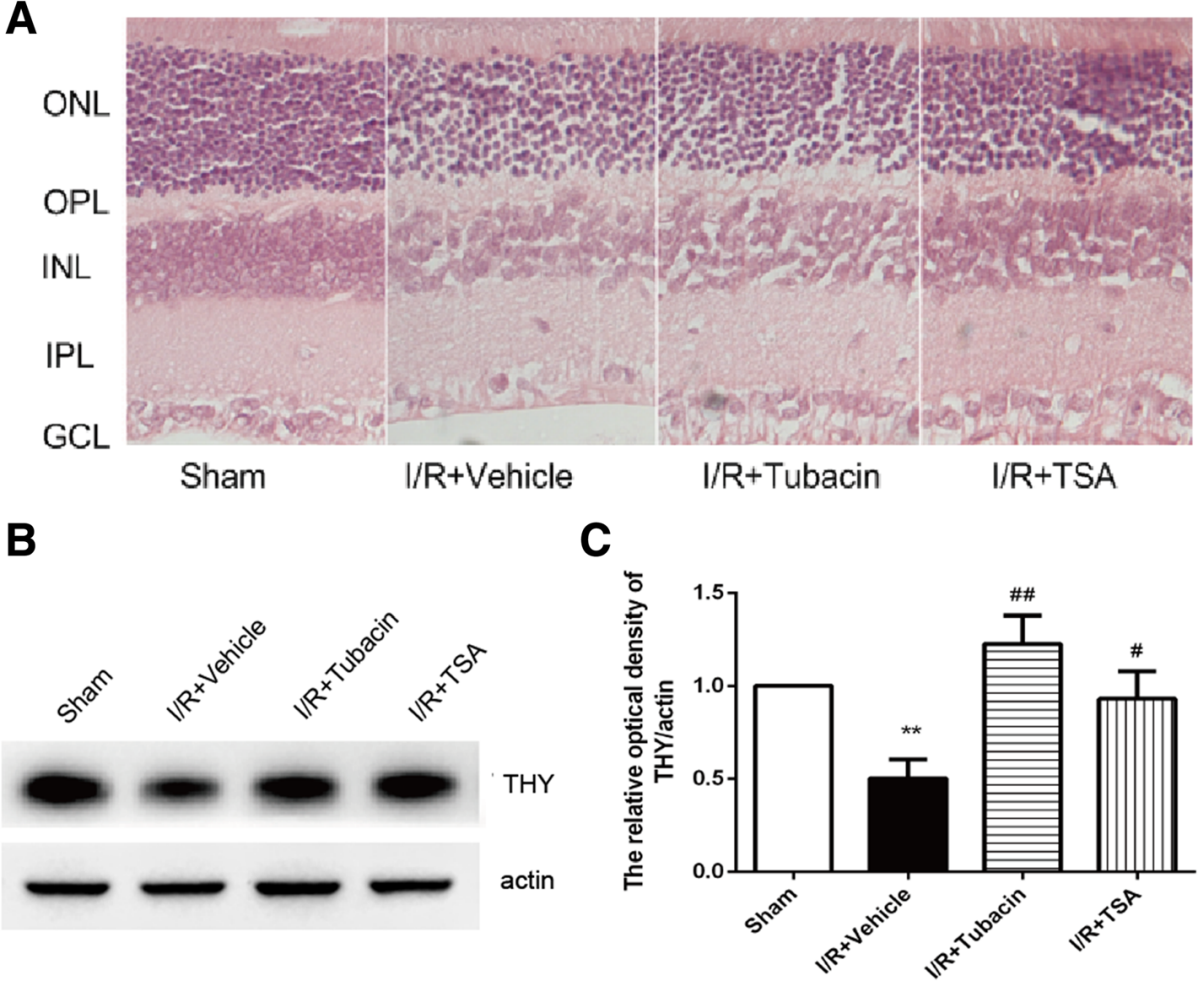
Effect of tubacin on retinal morphologic changes after I/R injury. (**a**) Photomicrographs of retinal cross-sections. Five well-organized retinal layers: ONL, outer nuclear layer; OPL, outer plexiform layer; INL, inner nuclear layer; IPL, inner plexiform layer; and GCL, ganglion cell layer. Analysis of the overall retinal thickness includes ONL, OPL, INL, IPL, and GCL. Analysis of the cell body counts from the retinal ganglion cell layer (GCL) over 200-μm distances. (**b**, **c**) Expression of THY as detected by Western blotting and quantitative analyses. The data are presented as means ± SEM (*n* = 5–8 rats per group). *P < 0.05, **P < 0.01 vs. Sham group; ^#^*P* < 0.05, ^##^*P* < 0.01 vs. I/R-plus-vehicle

The morphological changes induced by 50-min ischaemia in each group were assessed on days 3 after I/R injury (Fig. 1a; Table 2). Table 2 showed the overall retinal thickness in the I/R-plus-vehicle group had significantly decreased at 3 days after I/R injury compared with that in the sham-operated group. The inner nuclear layer (INL) thickness and inner plexiform layer (IPL) thickness in the I/R-plus-vehicle group were both reduced. A significantly decreased cell count in the I/R-plus-vehicle group was observed in the ganglion cell layer (GCL) that was reduced by 26.2% compared with that in the sham-operated group. By contrast, the overall retinal thickness in the I/R-plus-tubacin group or in the I/R-plus-TSA group was significantly increased compared with that in the I/R-plus-vehicle group. Tubacin or TSA could significantly restore the IPL thickness. The loss of the GCL cells was restored only by tubacin, while the INL thickness was recovered only by TSA.

**Table 2:** Effect of tubacin on ischaemia-induced depth of overall retina, inner retina, and number of cells in GCL

	Overall retinal thickness (μm)	Inner nuclear layer (μm)	Inner plexiform layer (μm)	Number of cells in GCL (per mm)
Sham	300.56 ± 61.82	49.01 ± 7.94	69.98 ± 15.25	42.65 ± 1.68
I/R + Vehicle	224.44 ± 25.33^**^	41.64 ± 8.04^*^	45.95 ± 11.85^**^	31.47. ± 1.47^**^
I/R + Tubacin	283.89 ± 54.12^##^	44.76 ± 4.37	62.15 ± 18.20^##^	37.04 ± 1.66^#^
I/R + TSA	287.22 ± 51.56^##^	49.02 ± 5.89^##^	71.58 ± 8.60^##^	34.39 ± 1.30

At 3 days after I/R, the eyes were enucleated, and cross-sections were prepared. Retinal I/R induced a significant decrease in the overall retinal thickness from GCL to ONL, inner retina thickness including INL and IPL, and cell number of GCL. The alteration in overall retinal thickness, inner retina including INL and IPL, and in cell number of GCL was partially prevented by tubacin. The values are expressed as means ± SEM (*n* = 5–8 rats per group). **P < 0.01 vs. Sham; ^#^*P* < 0.05 vs. I/R + vehicle

Additionally, Western blotting (Fig. 1b, c) results demonstrated that I/R-induced down-regulation of protein expression of THY gene, a ganglion cell-specific marker in rodent retina, could be antagonized by tubacin. These above results demonstrated that HDAC6 is involved in I/R-induced retinal morphological injury.

### HDAC6 expression and HDAC activity in normal or I/R injury retinas

**Fig. 2:**
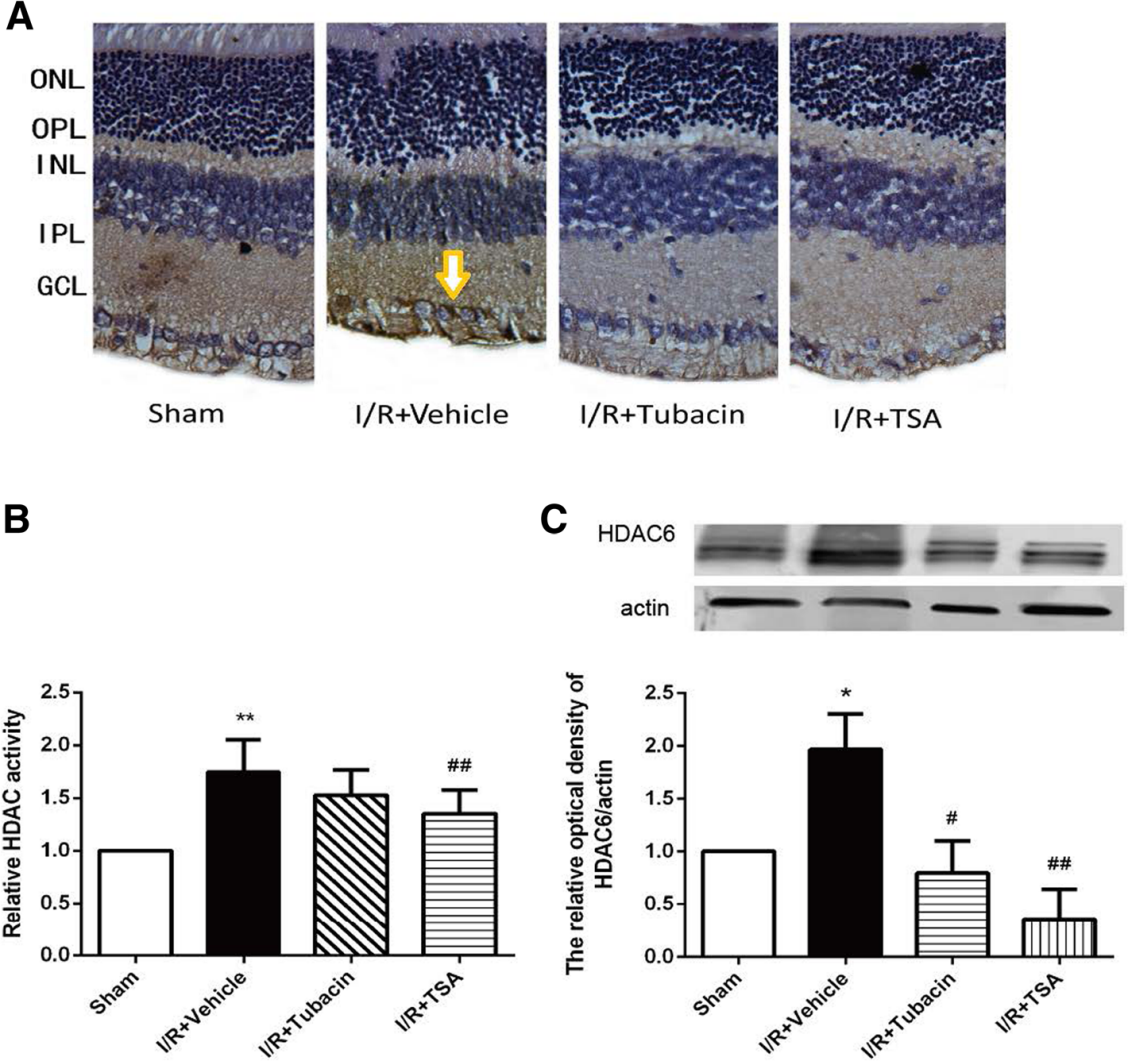
HDAC6 expression and effect of tubacin in the retina. (**a**) Immunohistochemical staining of HDAC6 in the retina (brown). HDAC6 immunoreactivity in the sham-operated retina, I/R-plus-vehicle retina, I/R-plus-tubacin retina and I/R-plus-TSA retina. (**b**) HDACs activity of retinas in different group. (**c**) Western blotting analysis of HDAC6 expression in retinal lysates. The values are expressed as means ± SEM (*n* = 5–8 rats per group). *P < 0.05, **P < 0.01 vs. Sham group; ^#^*P* < 0.05, ^##^*P* < 0.01 vs. I/R-plus-vehicle

Immunohistochemical staining revealed that HDAC6 is primarily localized in the INL and GCL, with little expression in the ONL, OPL and IPL (Fig. 2a). HDAC activity in normal retinas was low but was increased significantly after I/R injury. The increased retinal HDAC activity after retinal I/R injury can be attenuated either by the pan-HDAC inhibitor TSA (26.5%) or HDAC6 inhibitor tubacin (20.7%) compared with that in the I/R group (Fig. 2b). Immunohistochemical staining (Fig. 2a) and Western blot analysis (Fig. 2c) revealed that HDAC6 expression was significantly increased after I/R injury compared with that in the sham-operated retinas. The high expression of HDAC6 after retinal I/R injury could be attenuated either by TSA or tubacin.

### Effect of tubacin on retinal cell apoptosis and autophagy after I/R injury

**Fig. 3:**
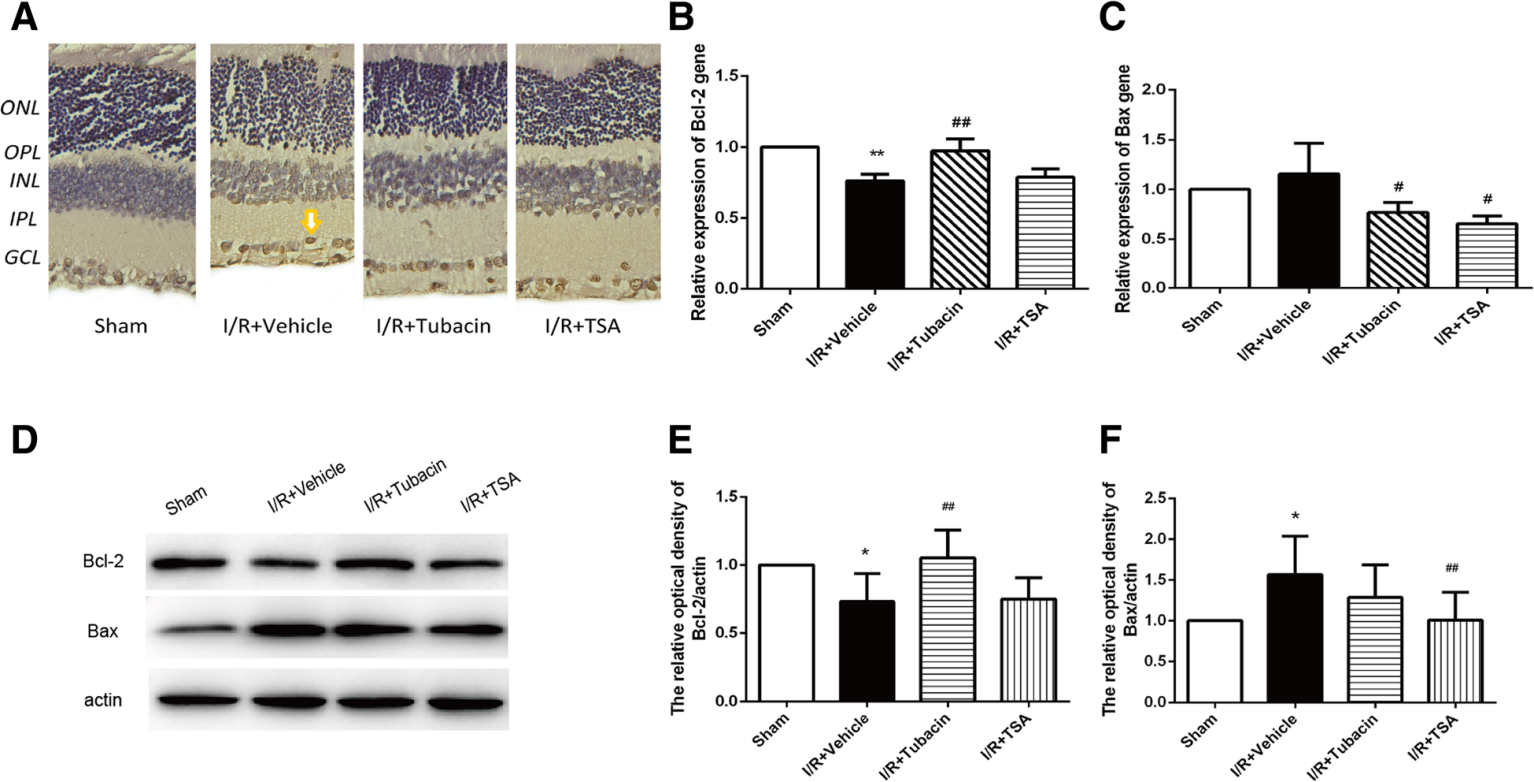
Effect of tubacin on apoptosis-related genes or protein expression in the retina after I/R injury. (**a**) TUNEL staining was performed on sections from the retina. Sham; Vehicle+I/R; I/R + tubacin; I/R + TSA. Few TUNEL-positive cells were observed in the sham group, abundant brown nuclei were observed in the I/R-plus-vehicle rats, and few brown nuclei were found in the I/R-plus-tubacin retinas. (**b**, **c**) RT-PCR analysis of Bcl-2 and Bax. (**d**) The expression of Bcl-2 and Bax was determined by Western blotting. (**e**, **f**) Quantitative analysis of the expression of Bcl-2 and Bax. The data are presented as means ± SEM (*n* = 5–8 rats per group). * *P* < 0.05, ** *P* < 0.01 vs. Sham group; ^#^*P* < 0.05, ^##^*P* < 0.01 vs. I/R-plus-vehicle

Because cell apoptosis and autophagy are the prominent features of axonal degeneration in the optic nerve, we next evaluated whether HDAC6 is involved in the regulation of cell apoptosis and autophagy after I/R injury. The apoptotic cells were counted by the TUNEL assay (Fig. 3a). The number of TUNEL-positive cells in the I/R-plus-vehicle group was obviously higher than that in the sham group. However, a decrease occurred in the density of apoptotic cells in the I/R-plus-tubacin group compared with that in the I/R-plus-vehicle group. This result suggested that I/R-induced apoptotic cell death can be alleviated by the HDAC6 inhibitor tubacin in the rat model.

RT-PCR (Fig. 3b, c) and Western blotting (Fig. 3d, e, f) showed that I/R injury activated Bax and attenuated the expression of Bcl-2. By contrast, tubacin significantly inhibited the activation of Bax and increased the expression of Bcl-2. These data suggested that tubacin suppressed I/R injury-induced retinal cell apoptosis.

**Fig. 4:**
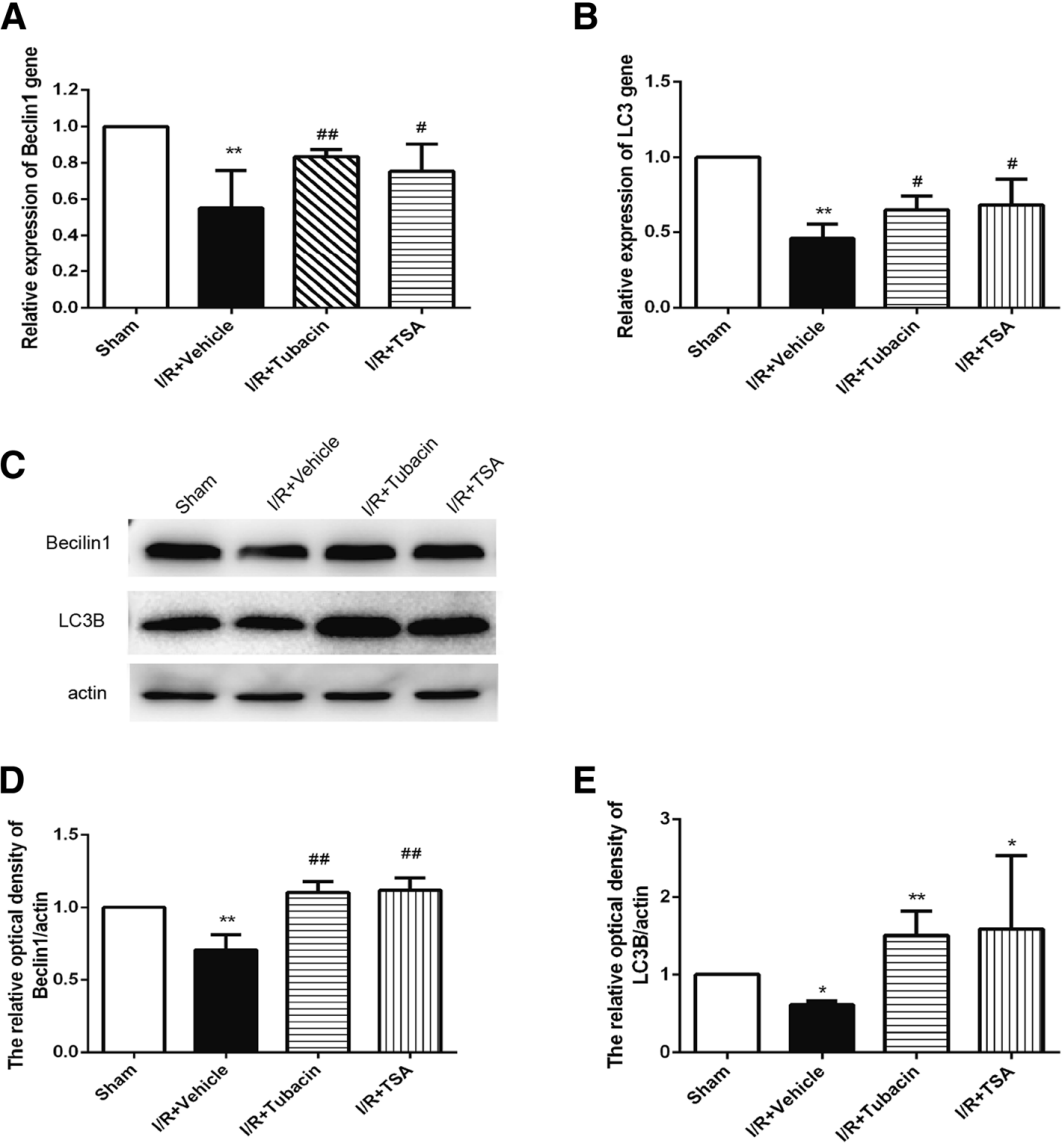
Effect of tubacin on autophagy-related genes after I/R injury. (**a**, **b**) RT-PCR analysis of LC3B and Beclin1 expression in the retina of different groups. (**c**, **d**, **e**) The expression of LC3B and Beclin1 was determined by Western blotting. The data are presented as means ± SEM (*n* = 5–8 rats per group). *P < 0.05, **P < 0.01 vs. Sham group; ^#^*P* < 0.05, ^##^*P* < 0.01 vs. I/R-plus-vehicle

The expression of autophagy-related genes and proteins were evaluated by RT-PCR (Fig. 4a, b) and Western blotting (Fig. 4c, d, e). The levels of LC3B and Beclin1 were decreased in the retina of the I/R-plus-vehicle group, indicating autophagy was diminished after I/R. However, the levels of LC3B and Beclin1 in the retina could be significantly upregulated by pretreatment with tubacin. These results demonstrated that the I/R injury-induced autophagy activity could be reversed by attenuating HDAC6.

### Effect of tubacin on increasing Prx2 after I/R injury

**Fig. 5:**
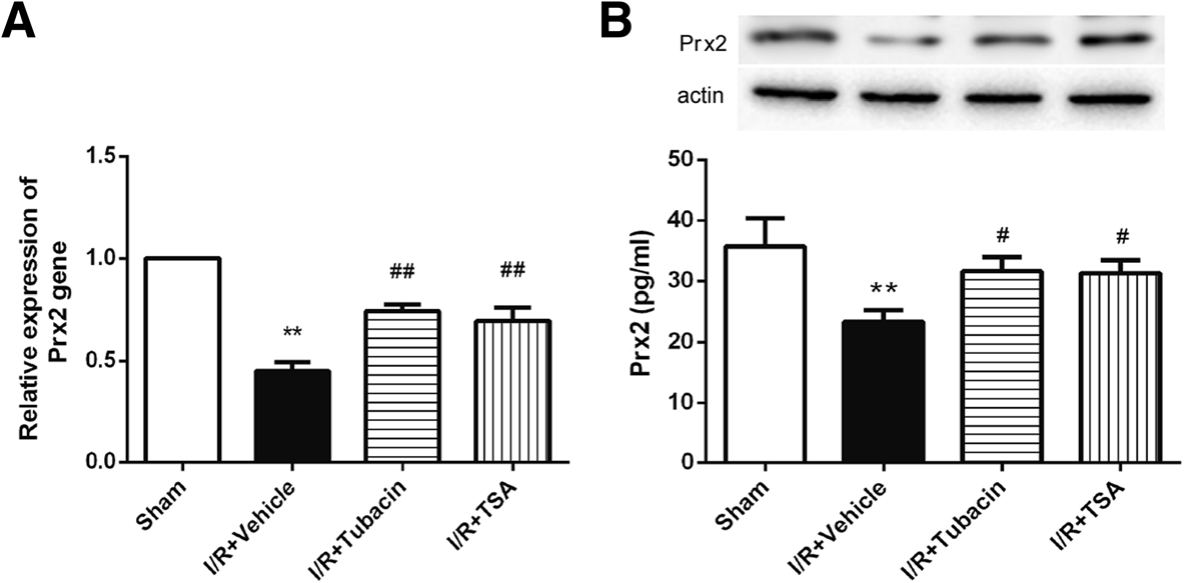
Effect of tubacin on Prx2 after I/R injury. The animals were treated with vehicle or tubacin, and Prx2 was measured 8 h after ischaemia. (**a**) PCR analysis of Prx2 expression. (**b**) Prx2 level detected by ELISA. The data are expressed as means ± SEM. **P < 0.01 vs. Sham group; ^#^*P* < 0.05, ^##^*P* < 0.01 vs. I/R-plus-vehicle

Several studies have suggested that Prx2 is related to the development of neurodegenerative disease and confirmed that Prx2 is a specific target of HDAC6; thus, we observed the expression of Prx2 in the retinal I/R injury animal model and role of HDAC6. RT-PCR (Fig. 5a) and ELISA (Fig. 5b) analyses revealed that Prx2 was significantly decreased after I/R, whereas TSA, as well as tubacin, could reverse I/R-induced events that decrease Prx2. These results indicated that manipulating HDAC6 by tubacin could increase the expression of Prx2 influenced by I/R injury.

### Effect of tubacin on restoring the α-tubulin acetylated levels

**Fig. 6:**
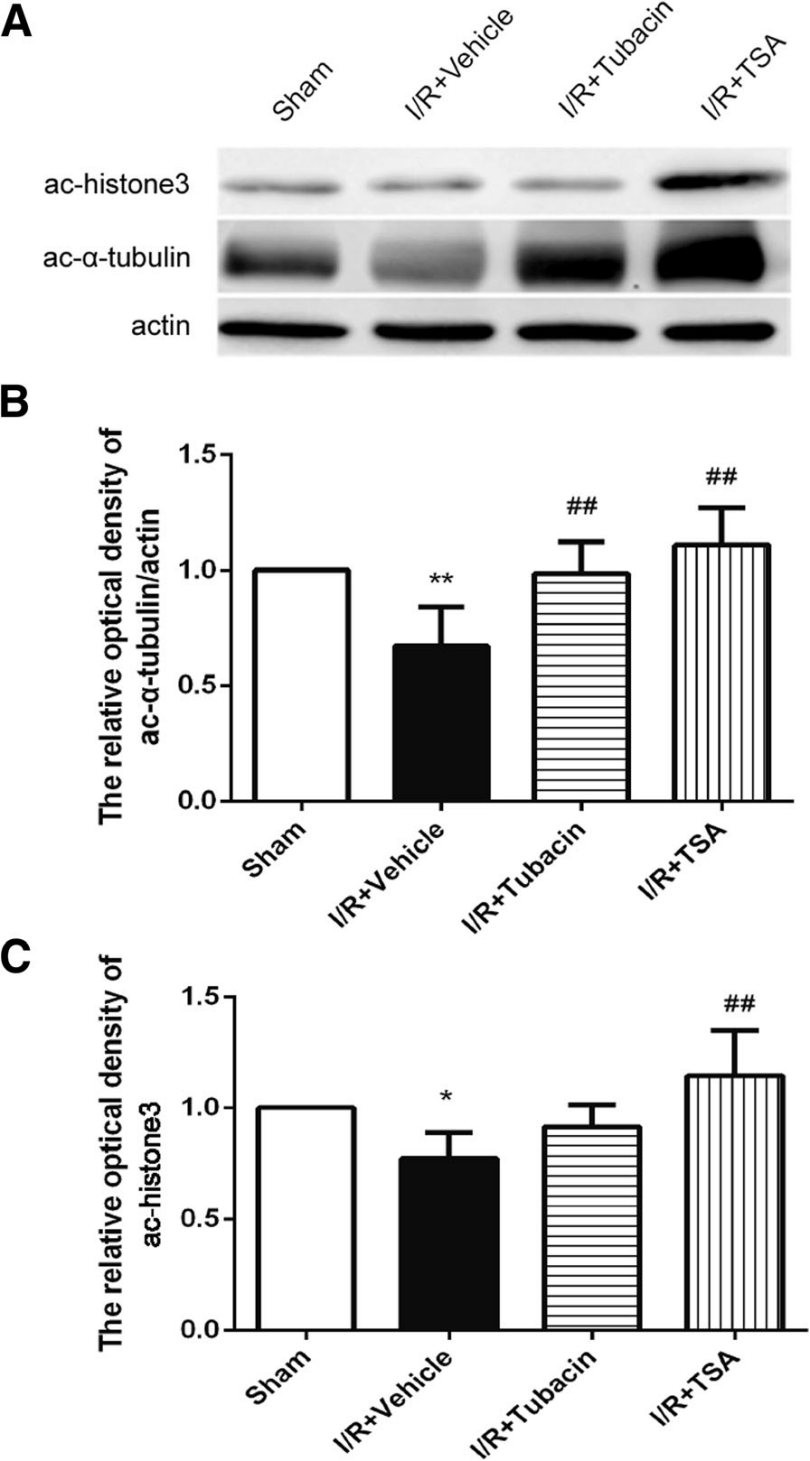
Effect of tubacin on the retinal acetylation of histone 3 and α-tubulin after I/R injury. (**a**) The expression of acetylated α-tubulin and histone 3 in the retina as determined by Western blotting. (**b**, **c**) Quantitative analysis of the expression of acetylated α-tubulin and histone 3. *P < 0.05, **P < 0.01 vs. Sham group; ^##^*P* < 0.01 vs. I/R-plus-vehicle

To evaluate whether treatment with tubacin could result in the hyperacetylation of retinal proteins, the expression of acetylated histone-3 in the retina was determined by Western blot analysis. The result showed that the level of acetylated histone-3 in the retinas of tubacin-treated animals was relatively lower than that of the sham group. However, the level of retina histone-3 acetylation in the TSA-treated group was markedly increased (Fig. 6a, c). The relatively high expression of acetylated α-tubulin was detected in the retinas of both tubacin-treated and TSA-treated animals (Fig. 6a, b). These results indicated that the neuroprotection of tubacin did not involve the histone acetylation but tubulin stabilization.

## Discussion

It was confirmed that the HDAC6 inhibitor tubacin could protect the retina from I/R injury, as indicated by the preservation of retinal morphology and neurons in the GCL. Moreover, tubacin pretreatment inhibited the expression of pro-apoptotic proteins and inflammatory mediators and enhanced the expression levels of Bcl-2, which inhibits apoptosis. Tubacin treatment also upregulated the expression of autophagy-related proteins and anti-oxidative proteins. Furthermore, the protective role of tubacin was associated with acetylated α-tubulin rather than acetylated histone. These results demonstrated that HDAC6 plays an important role in I/R-induced neurodegenerative disorders of the retina.

Until now, eighteen human HDACs of four categories were discovered, the functions of are involved in apoptosis, differentiation, the cell cycle and transcription. HDAC1, HDAC2, HDAC3 and HDAC8 in Class I are distributed ubiquitously, while HDAC4, HDAC 5, HDAC6, HDAC7, HDAC9 and HDAC10 in class II are distributed specifically. HDAC6 belongs to class IIb HDACs and is unique in that it is a cytoplasmic microtubule-associated enzyme. HDAC6 deacetylates tubulin, HSP90, and cortactin, forms complexes with other partner proteins and is involved in numerous biological processes, such as cell migration and cell-to-cell interactions [17, 20, 24, 25]. Preliminary studies on the retina of mice proved the expression of HDAC1 3, 4, 5, 6, 16, and 17. These isoforms play essential roles in the development of Müller cells and neurocytes in the retina that include ganglia and bipolar and rod cells. Our present study agreed with these studies and showed that HDAC6 is expressed in the cytosol of the retina and is mainly localized in the inner retinal layer and GCL. Furthermore, I/R injury significantly increased the expression of HDAC6, which could be inhibited by the selective HDAC6 inhibitor tubacin and pan-HDAC inhibitor TSA. Our study suggests that HDAC6 may be involved in I/R-induced retinal neurodegeneration, and inhibiting HDAC6 may exert a neuroprotective effect.

Glaucoma is a primary cause of global blindness. The disease is characterized by the progressive degeneration of RGCs and their axons, resulting in irreversibly damaged visual function in the end [26, 27]. All the programmed cell death (PCD) types, including apoptosis and autophagy, play important roles in the development of the glaucomatous retina of glaucoma patients and mammalian models. It is crucial to better understand the molecular mechanism of retinal PCD and develop better therapies. The methods aimed at understanding the molecular mechanisms of PCD and its regulation may be helpful for neuronal survival and preservation of visual function. The understanding of the potential principle of the deaths of retinal neurons in glaucoma are probably increased; thus, the relevant therapies are improved by using the model of I/R injury in the retina, mimicking the clinical manifestations of acute angle closure glaucoma. Our study also indicated that the HDAC6 inhibitor tubacin could attenuate I/R-induced RGC loss.

Autophagy is an evolutionarily conserved process that is involved in regulating organelles and proteins [28]. It plays a key role in maintaining intracellular homeostasis [29] and cell survival in a stressful environment. According to different circumstances, autophagy can have beneficial or deleterious effects on neurons [3]. Thus far, little is known about the role of the autophagic pathway in RGC death. Activation of autophagy was investigated in RGCs following optic nerve transection, and a protective role was indicated in RGC-5 cells in starvation [18]. The persistent accumulation of autophagosomes, concurrent with injury-induced axonal degradation and secondary to lesion-induced calcium influx, was reported in the optic nerve following optic nerve crushing.

Presently, the role of autophagy in I/R injury remains controversial. The effect of inhibiting autophagy was considered as protection for neurocytes and potentially as an innovative approach to preventing ischaemia injuries. Nevertheless, activated autophagy was also considered as protective for neurocytes in the ischaemic brain and I/R retina [3]. Our results support the view that autophagy plays a protective role in retinal I/R. Beclin1 plays an important role in the induction of autophagy, and LC3 is involved in the maturation process of autophagosomes. A significant decrease in Beclin1 and LC3 expression was observed in retinas subjected to I/R injury, suggesting impaired autophagic activity that seemingly agreed with the conclusion of a previous study [3].

Additionally, the multifarious effect of HDAC6 on stimulating autophagy was unrevealed yet and disputable to a certain extent. Despite abundant facts indicating the stimulative role of HDAC6 in autophagic activity, the latest results suggested the necessity of hyper-acetylated α-tubulin in generating an assembly platform for autophagosomes [30–32]. HDAC6 is indispensable to fusing autophagosomes and lysosomes instead of causing autophagy [33]. The significance of HDAC6 in selective autophagy was probably superior to that in other types of autophagy—for example, autophagy caused by starvation—because HDAC6 has domains that bind ubiquitin and could modulate the responses to unfolded proteins [33]. Our results also showed that tubacin administration increased the expression levels of retinal Beclin1 and LC3B after I/R injury, indicating that tubacin reversed retinal autophagic activity, which was probably related to the principle of tubacin preventing I/R injury. We noted that the observed increase in autophagy may be traced back to the inhibition of HDAC6-mediated tubulin deacetylation [34]. Further research is needed to clarify the mechanism of the regulation of tubacin in the autophagy activity of the retina and to identify new targets for autophagy pathways to establish new treatment strategies for I/R injury.

RGC deaths induced by I/R were postponed by tubacin treatment, a finding that agreed with the results of former studies that tubacin effectively protected neurons in other neurodegenerative disorders of the central nervous system [35, 36]. However, recent studies on cancer therapy have demonstrated that HDAC6 inhibitors inhibit proliferation and induce apoptosis in multiple myeloma cells. This consistency presumes that tubacin could indirectly exert anti-apoptotic activity via HSP90 or Prxs. This protective effect was accompanied by tubacin-mediated inhibition of a pro-apoptosis molecule (Bax) and induction of anti-apoptotic factors (Bcl-2). Bcl-2 is associated with apoptosis [37] and can be induced via various stimuli, including glucose deprivation, growth factor deprivation and lipid peroxidation [38]. The induction of Bcl-2 has been indicated to protect against apoptosis [39]. Bax, a pro-apoptotic protein of the Bcl-2 family of proteins, could strongly facilitate mitochondrial membrane permeabilization and the activation of nucleases and caspases. This can result in irreversible damage to the mitochondria and acceleration of programmed cell death [40, 41]. The anti-apoptosis effect of tubacin was confirmed by the attenuation of cell death due to I/R in this study.

Prxs belong to a superfamily of thiol peroxidases that act as the catalyst in the reactions of reducing reactive oxygen species (ROS) and consequently play a crucial role in oxidation resistance and redox signalling pathways. Prx2 is especially essential to protect the nerve system, and it has cytoprotective effects in response to transient brain ischaemia and oxidative stress [42–45]. Parmigiant et al. reported that HDAC6 is a specific deacetylase of Prxs and is involved in redox regulation [46]. Previous studies have revealed that, after being intensively exposed to ROS, cysteine in the active sites of Prx2 was hyper-oxidized into derivatives of cysteine sulfonic acid or sulfinyl (Cys-SO2/3) and the activity of peroxidase for protection was lost [47, 48]. Our study agrees with the above studies. HDAC6 and Prxs may be targets to modulate the intracellular redox status in neurodegenerative diseases, and the HDAC6 inhibitor exerted retinal neuroprotection due to I/R injury.

## Conclusions

Our studies demonstrate that pretreatment with the HDAC6 inhibitor against I/R injury in the retina may exert potential retinal neuroprotection by enhancing autophagy, inhibiting apoptosis, and modulating anti-oxidative stress. Furthermore, its protective effect may be independent of histone acetylation and mediated by acetylated tubulin. These findings support the view that the regulation of acetylation in the retina is not only histone dependent but also histone-independent regulation, with potential beneficial effects as a neuroprotectant. Our findings may provide a potential neuroprotective strategy for the treatment of glaucoma.

## Abbreviations

ABC: Avidin-biotin-perosidase complex
Bax: Bcl-2 Assaciated X protein
Bcl-2: B-cell lymphoma-2
Cys-SO2/3: cysteine sulfonic acid or sulfinyl
DAB: Diaminobenzidine
GCL: Ganglion cell layer
H&E: Hematoxylin and eosin
HDAC: Histone deacetylase
HSP: Heat Shock Proteins
I/R: Ischemia and reperfusion
INL: Inner nuclear layer
IOP: Intraocular pressure1
IPL: Inner plexiform layer
LC3: light chain 3
ONH: Optic nerve head
ONL: Outernuclear layer
OPL: Outer plexiform layer
PCD: Programmed cell death
POD: Peroxidase
Prx2: Peroxiredoxin 2
RGC: Retinal ganglion cell
ROS: Reactive oxygen species
TSA: Trichostatin A
VPA: Valproic acid
